# The Feasibility of Combining ADC Value With Texture Analysis of T_2_WI, DWI and CE-T_1_WI to Preoperatively Predict the Expression Levels of Ki-67 and p53 of Endometrial Carcinoma

**DOI:** 10.3389/fonc.2021.805545

**Published:** 2022-01-20

**Authors:** Xueyan Jiang, Haodong Jia, Zhongyuan Zhang, Chao Wei, Chuanbin Wang, Jiangning Dong

**Affiliations:** ^1^ Department of Radiology, Bengbu Medical College, Bengbu, China; ^2^ Department of Radiology, The First Affiliated Hospital of the University of Science and Technology of China, Anhui Provincial Cancer Hospital, Hefei, China

**Keywords:** endometrial carcinoma, p53, Ki-67, apparent diffusion coefficient, texture analysis

## Abstract

**Purpose:**

To evaluate the feasibility of apparent diffusion coefficient (ADC) value combined with texture analysis (TA) in preoperatively predicting the expression levels of Ki-67 and p53 in endometrial carcinoma (EC) patients.

**Methods:**

Clinical, pathological and MRI findings of 110 EC patients were analyzed retrospectively. The expression levels of Ki-67 and p53 in EC tissues were detected by immunohistochemistry. ADC value was calculated, and three-dimensional (3D) texture features were measured on T_2_-weighted images (T_2_WI), diffusion-weighted images (DWI), and contrast-enhanced T_1_-weighted images (CE-T_1_WI). The univariate and multivariate logistic regression and cross-validations were used for the selection of texture features. The receiver operating characteristic (ROC) curve was performed to estimate the diagnostic efficiency of prediction model by the area under the curve (AUC) in the training and validation cohorts.

**Results:**

Significant differences of the ADC values were found in predicting Ki-67 and p53 (*P*=0.039, *P*=0.007). The AUC of the ADC value in predicting the expression levels of Ki-67 and p53 were 0.698, 0.853 and 0.626, 0.702 in the training and validation cohorts. The AUC of the TA model based on T_2_WI, DWI, CE-T_1_WI, and ADC value combined with T_2_WI + DWI + CE-T_1_WI in the training and validation cohorts for predicting the expression of Ki-67 were 0.741, 0.765, 0.733, 0.922 and 0.688, 0.691, 0.651, 0.938, respectively, and for predicting the expression of p53 were 0.763, 0.805, 0.781, 0.901 and 0.796, 0.713, 0.657, 0.922, respectively.

**Conclusion:**

ADC values combined with TA are beneficial for predicting the expression levels of Ki-67 and p53 in EC patients before surgery, and they provide higher auxiliary diagnostic values for clinical application.

## Introduction

Endometrial carcinoma (EC) is one of the most common malignancies of the female reproductive system worldwide (1), and the morbidity and mortality of EC have been rising with a trend towards a younger age (2). It has been suggested that the occurrence and development of EC are related not only to estrogen levels but also to the proliferation and apoptosis of tumor cells (3). A necessary condition for normal functioning of the body is to maintain the dynamic balance between cell proliferation and apoptosis (4, 5). If this balance is broken, it will promote the occurrence of tumors. Ki-67 and p53 are closely related to the proliferation and apoptosis of tumor cells. Ki-67 is a marker of cell proliferation, is mainly expressed in the nucleus of proliferating cells, and is used to evaluate the proliferation state of tumor cells (6). Higher values of Ki-67 indicate increased malignancy and invasiveness of tumours (7). p53 is an important tumor suppressor gene that controls the initiation of the cell cycle, regulates cell division, inhibits cell growth, regulates transcription, and induces apoptosis (8).

DWI determines the cell density of tissues by detecting the diffusion of water molecules and quantifying it by using the ADC value (9). It has been shown (10) that the ADC mean value is related to the expression level of Ki-67 and p53 in esophageal squamous cell carcinoma, which can be used as a noninvasive biological indicator to predict the proliferation of esophageal squamous cell carcinoma cells and to determine the prognosis of patients. TA is a method to quantitatively measure the distribution and (or) relationship between histogram, pixel intensity or grey level of an image in the region of interest (11, 12). TA highlights subtle patterns of tissue distribution (texture features) that cannot be recognized by human eyes and extends the intrinsic value of images. Thus, in recent years, TA has been used with various cross-sectional imaging modalities and shows clinical applicability in the detection, diagnosis, prognosis, characterization and response evaluation of different cancers (13–15). In the present study, five models were developed to preoperatively predict the expression levels of Ki-67 and p53 in EC. The purpose of this study was to noninvasively evaluate the expression levels of Ki-67 and p53 in EC and to provide imaging markers for the clinical diagnosis and treatment of EC.

## Materials and Methods

### Patients

**Figure 1:**
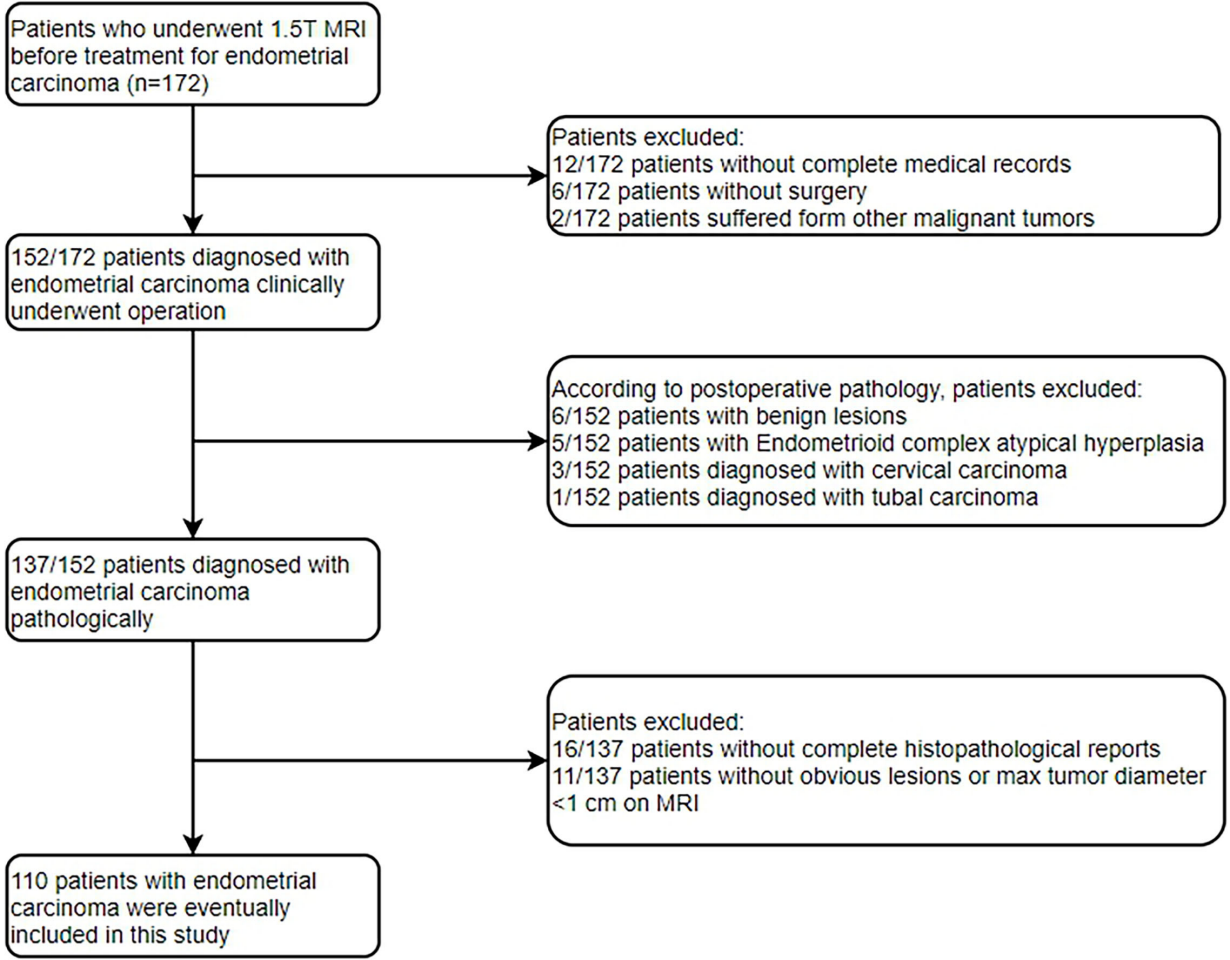
Flowchart shows selection of studying population and exclusion criteria.

The Institutional Review Board approved the present retrospective study, and informed consent was waived. Between January 2015 and December 2020, 172 patients who underwent conventional 1.5T MRI before surgery were enrolled by searching our pathology and radiology database in the study. The patients were screened through the medical record system of our hospital. The inclusion criteria were as follows: (a) histologic confirmation of primary EC according to the World Health Organization criteria; (b) no history of preoperative treatment; (c) preoperative available T_2_WI, DWI and CE-T_1_WI images; (d) available Ki-67 and p53 expression based on immunohistochemical detection; (e) lesion that could be measured and segmented on MRI. Patients were excluded for the following reasons: (a) incomplete medical records or did not receive treatment in our hospital (n=18); (b) other malignant tumors (n=2); (c) postoperative pathology of non-endometrial carcinoma (n=15); (d) inadequate histopathological reports (n=16); (e) without obvious lesions or maximal tumor diameter of less than 1 cm on MRI (n=11). Finally, 110 patients were included in the present study. The flowchart of the exclusion criteria is shown in **Figure 1**.

Patients were divided into a training cohort and a validation cohort at a ratio of 7:3. This division was based on the chronological order in which patients were examined to ensure the randomness of the pathological results in the training and validation cohorts. The prediction models of Ki-67 and p53 were built with a training cohort and evaluated with a validation cohort. In the p53 expression of EC, only 38 patients with negative p53 expression and 72 patients with positive p53 expression were enrolled. The ratio of negative p53 expression patients to positive p53 expression patients was about 1:1.89, revealing a sample imbalance. Therefore, the synthetic minority over-sampling technique (SMOTE) algorithm was used to balance the minority class, so that the two classes of EC patients were 1:1(72 negative p53 expression and 72 positive p53 expression).

### MR Imaging

All MR imaging studies were performed with 1.5T MR imaging units (United Imaging Healthcare, uMR560, Shanghai) using a 6-channel body array coil. All patients were asked to fast for at least 4 hours, and intramuscular injection of scopolamine butyl bromide was given half an hour before the MRI examination to reduce bowel peristalsis. Detailed scanning parameters are listed as follows: (1) Axial fast spin echo (FSE) T_1_-weighted images (T_1_WI): repetition time (TR)/echo time (TE): 391 ms/9.3 ms, slice thickness: 6 mm, inter-slice gap: 2 mm, a field of view (FOV): 38 cm × 28 cm, and matrix size: 320 × 224. (2) Axial FSE T_2_-weighted images (T_2_WI): TR/TE: 4500 ms/93 ms, slice thickness: 6 mm, inter-slice gap: 2 mm, FOV: 28 cm × 25 cm, and matrix size: 320 × 224. (3) Axial DWI with b-values of 0 and 1000 s/mm^2^: TR/TE: 3201 ms/75.4 ms, slice thickness: 6 mm, inter-slice gap: 2 mm, FOV: 38 cm× 28 cm, and matrix size: 128 × 128. (4) Three-dimensional volumetric interpolated quick gradient echo contrast-enhanced imaging on axial: TR/TE: 8 ms/3 ms, slice thickness: 5 mm, inter-slice gap: 0 mm, FOV: 38 cm×28 cm, and matrix size 256×192. The image was exported in DICOM format.

### Image Analysis

**Figure 2:**
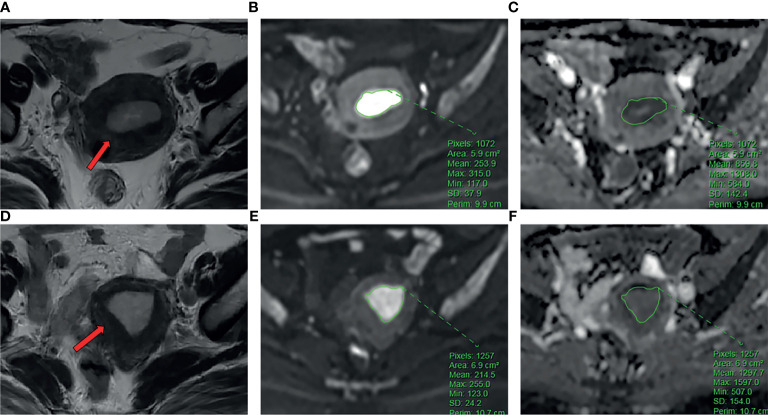
**(A-C)** The patient is a 54-year-old female with EC. Ki-67 expression was 70%, and p53 expression was positive. **(A)** Axial T_2_WI showed an irregular mass in the uterine cavity (arrow). **(B)** ROI of the largest lesion area on DWI. **(C)** The ADC values on ADC map. **(D–F)** The patient is a 58-year-old female with EC. Ki-67 expression was 60%, and p53 expression is negative. **(D)** Axial T_2_WI showed an irregular mass in the uterine cavity (arrow). **(E)** ROI of the largest lesion area on DWI. **(F)** The ADC values on ADC map.

For the measurement of ADC values, two subspecialty radiologists (reader 1 and reader 2) with 8 and 10 years of experience in imaging diagnosis independently examined the images to obtain ADC values. The original ADC data were directly analyzed in the postprocessing workstation (United Imaging Healthcare). Under the guidance of T_2_WI, the region of interest (ROI) was delineated in the solid part of the largest tumor layer on the DWI image with b=1000 s/mm^2^, avoiding hemorrhage and necrotic areas as much as possible. The averages were obtained after the repetition of the measurement three times. The ADC value extraction process is shown in **Figure 2**.

**Figure 3:**
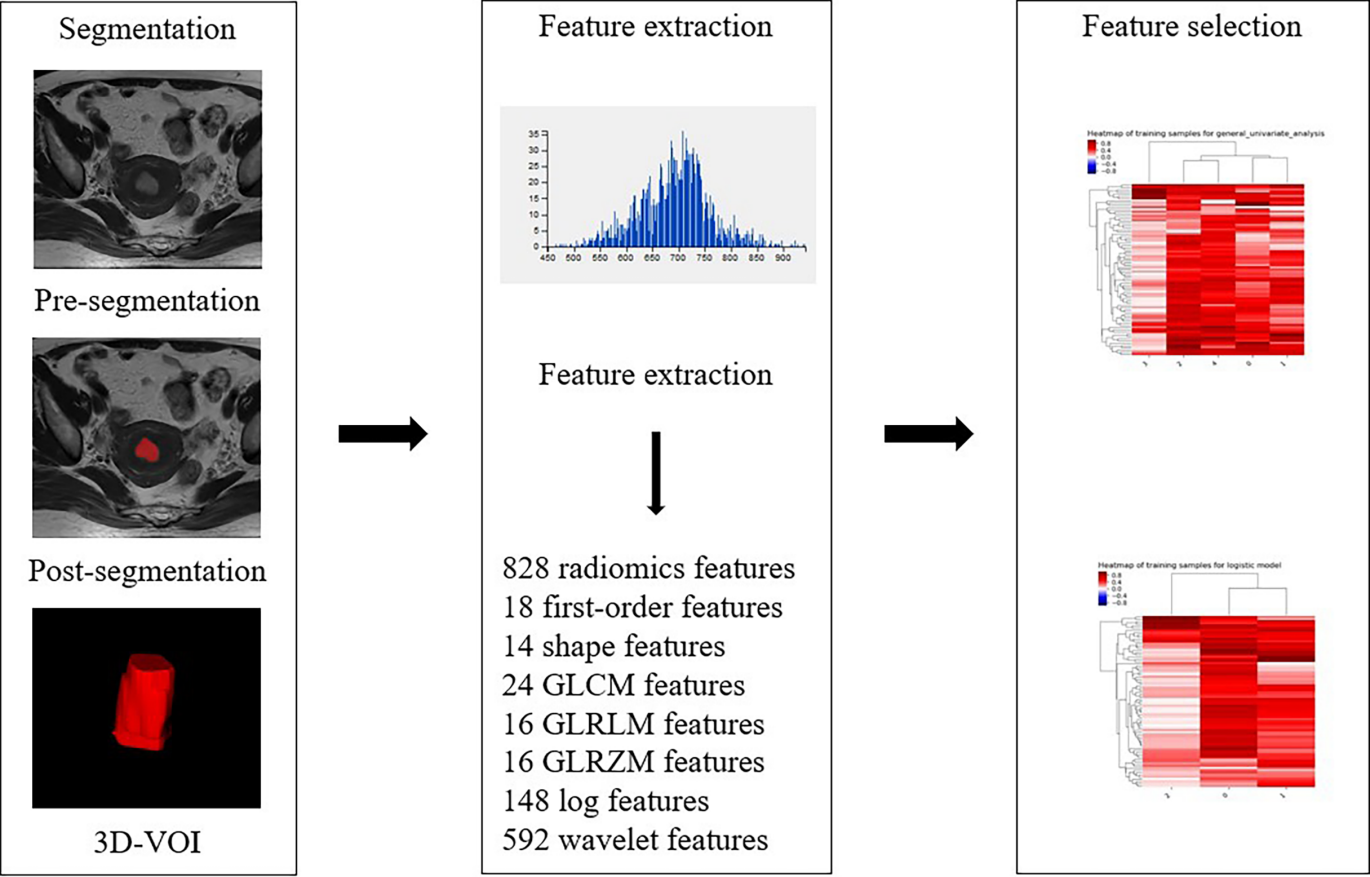
Radiomic workflow.

For the assessment of texture features, the conventional axial T_2_WI image, DWI with a b value of 1000 s/mm^2^, and axial CE-T_1_WI image were segmented by reader 2. The conventional axial T_2_WI image, DWI with a b value of 1000 s/mm^2^, and axial CE-T_1_WI image were imported into ITK-SNAP software (version 3.6.0, http://www.itksnap.org). The three-dimensional region of interest (3D ROI) of the whole tumor was manually delineated at each level of sequence, including areas of hemorrhage and necrosis site. To reduce registration errors, a DWI map with a b value of 1000 s/mm^2^ was used to assist segmentation. The 3D ROI file was then imported into AK (Analysis Kit, Kinetics Version 2.1, GE Health-care) software to extract texture features. Finally, a total of 828 texture features of the whole tumor were automatically extracted from each of the three MR scanning sequences. As an example, the texture feature extraction process on T_2_WI is shown in **Figure 3**. After one week, 30 patients were randomly selected, and the images of 30 patients were re-segmented by reader 1 and reader 2. The intraclass correlation coefficients (ICCs) were used to evaluate the stability of texture features between inter- and intrareader segmentations. The texture features with ICC > 0.75 were preserved.

### Pathological Examination

Postoperative Ki-67 and p53 testing were performed by two professional pathologists with more than 8 years of pathological diagnosis experience. The diagnostic criteria of immuno-histochemical staining for p53 included non-staining (0), faint staining (1+), moderate staining (2+) and strong staining (3+). 0 was viewed as negative expression, while 1+ ~ 3+ was viewed as positive expression. The expression of Ki-67 was localized in the nucleus of tumor cells, and five fields were randomly selected under the high-power microscope. The tumor cells were determined to be positive if there were brown particles in the cytoplasm of the tumor cells, and the staining intensity was higher than the background nonspecific staining. According to Li et al. (16), Ki-67 < 50% is the low expression group, and Ki-67 ≥ 50% is the high expression group.

### Statistical Analysis

The data were analyzed by SPSS 26.0 (IBM Corporation, Armonk NY, USA), IPMS (Version 2.4.0, GE Health-care) software, and MedCalc Statistical Software version 15.2.2 (MedCalc Software bvba, Ostend, Belgium). Data that had a normal distribution are expressed as the mean ± standard deviation, while abnormally distributed data are expressed as the median. Independent sample *t*-tests or Mann-Whitney *U*-tests were used to compare the ADC values and the texture features of each group. *P* < 0.05 was considered statistically significant. Univariate and multivariate logistic regression analyses and cross-validation were performed on the texture features with statistical significance between each subgroup to select the optimal texture features. The ADC model, TA model, and combined model were built in the training cohorts and validation cohorts to predict the expression levels of Ki-67 and p53. The ROC curves were plotted to assess the performance of the five models in both cohorts. The AUCs were compared using DeLong’s test of equality. *P* < 0.05 was considered as an independent predictor. A calibration curve was plotted to evaluate the agreement between the prediction result and gold standard.

## Results

### Clinical and Pathological Findings

Among the 110 EC patients, there were 50 cases of low Ki-67 expression and 60 cases of high Ki-67 expression as well as 38 cases of negative p53 expression and 72 cases of positive p53 expression. The age, FIGO stage, and histological type are presented in **Table 1**. Regarding Ki-67 expression, there was no significant difference between the two groups in age (*P*=0.319), but FIGO stage (*P*=0.020) and histological type (*P*<0.001) were significant differences. Regarding p53 expression, age (*P*=0.634) and FIGO stage (*P*=0.063) were not significantly different between the two groups, but histological type (*P*=0.030) was statistically significant.

**Table 1:** Patients clinical and pathological characteristics.

	Ki-67 (n=110)				p53 (n=110)			
	Patients (n)	Low expression (<50%)	High expression (≥ 50%)	*P-*value	Patients (n)	Negative expression (0)	Positive expression (1+ ~ 3+)	*P-*value
Total	110	50 (45.5%)	60 (54.5%)		110	38 (34.5%)	72 (65.5%)	
Age		53.6 ± 8.3^a^	55.3 ± 9.1	0.319^b^		54.0 ± 8.6	54.8 ± 8.9	0.634
FIGO^c^ stage								
I-II	97	48 (96.0%)	49 (81.7%)	0.020^d^	97	37 (97.4%)	60 (83.3%)	0.063
III-IV	13	2 (4.0%)	11 (18.3%)		13	1 (2.6%)	12 (16.7%)	
Histologic type								
^e^EMC	89	48 (96.0%)	41 (68.3%)	<0.001	89	35 (92.1%)	54 (75.0%)	0.030
^f^NEMC	21	2 (4.0%)	19 (31.7%)		21	3 (7.9%)	18 (25.0%)	

a Data are mean ± standard deviation.

b T-statistical test.

c FIGO, International Federation of Gynecology and Obstetrics.

d Chi-square test.

e EMC, Endometrioid carcinoma.

f NEMC, Non-endometrioid carcinoma.

### ICC Analysis of ADC Values

ICC analysis of measurements of ADC values by the two attending physicians showed good agreement between the surveyors [ICC = 0.883, 95% CI (0.825-0.921), *P*<0.001]. Therefore, this study used only the results of the first radiologist for a full-text analysis. The ADC values of subgroups were compared by independent sample *t*-test, and the results are shown in **Table 2**. The ADC values in EC with high Ki-67 expression were lower than that of the low Ki-67 expression (*P* =0.007 and *P*<0.001) with AUCs of 0.698 and 0.853 in the training and validation cohorts, respectively. The ADC value in EC with positive p53 expression was significantly lower than that in EC with negative p53 expression, and the difference was statistically significant (*P*=0.039 and *P*=0.048). The AUC of the ADC values in differentiating the level of p53 expression were 0.626 and 0.702 in the training and validation cohorts, respectively.

**Table 2:** The ADC value in relation to Ki-67 and p53 and its predictive performance.

	Training cohort				Validation cohort			
	n	ADC value×10^-3^mm^2^/s	*P*-value	AUC	*n*	ADC value×10^-3^mm^2^/s	*P*-value	AUC
low Ki-67 expression	34	0.933 ± 0.125	0.007	0.698	16	0.974 ± 0.131	<0.001	0.853
high Ki-67 expression	43	0.844 ± 0.150			17	0.772 ± 0.160		
negative p53 expression	28	0.929 ± 0.153	0.039	0.626	10	0.962 ± 0.183	0.048	0.702
positive p53 expression	49	0.858 ± 0.137			23	0.830 ± 0.163		

### Texture Feature Difference and TA Prediction Model of the Expression Level of Ki-67 and p53 in EC

A total of 828 texture features were obtained from AK software based on T_2_WI, DWI and CE-T_1_WI. Sufficient dimension reduction was obtained with the use of univariate and multivariate logistic regression analysis and cross-validation in the training cohort. Three texture features based on T_2_WI, DWI, and CE-T_1_WI were extracted from Ki-67 expression, and the detailed information for these texture features is listed in **Table 3**. Four texture features based on T_2_WI, DWI, and CE-T_1_WI were extracted from p53 expression, and the detailed information for these texture features is listed in **Table 4**. The AUC, sensitivity, and specificity of the models that MR imaging-based TA used to predict the expression level of Ki-67 and p53 in the training and validation cohorts are shown in **Table 5**.

**Table 3:** Statistical results of texture features of Ki-67 high and low expression groups in EC.

Texture features	low Ki-67 expression	high Ki-67 expression	Multivariate logistic regression analysis		AUC
			OR	*P*	
T_2_WI- texture features					
T_2_WI-wavelet-HLL_firstorder_Skewness	-0.805 ± 0.622	-0.399 ± 0.440	48.597	0.011	0.701
T_2_WI-wavelet-LLL_firstorder_Minimum	1125.378 ± 359.711	1277.809 ± 309.657	24.502	0.024	0.648
T_2_WI-wavelet-HHL_glszm_SZN	2.867(1.782,3.522)	2.833(1.800,5.870)	14.557	0.047	0.530
DWI- texture features					
DWI-glcm_Correlation	0.212 ± 0.225	0.312 ± 0.202	14.134	0.020	0.646
DWI-wavelet-HHL_glszm_HGLZE	1.757 ± 0.333	1.969 ± 0.362	40.908	0.008	0.662
DWI-wavelet-LHL_firstorder_IR	3.006 ± 1.488	3.690 ± 1.633	49.623	0.017	0.669
CE-T_1_WI- texture features					
CE-T_1_WI-wavelet-HLL_glszm_SAE	0.315 ± 0.091	0.339 ± 0.088	20.763	0.042	0.625
CE-T_1_WI-wavelet-HLH_firstorder_Kurtosis	4.503 ± 1.212	3.924 ± 0.684	0.037	0.018	0.647
CE-T_1_WI-wavelet-LLH_glcm_Correlation	0.251 ± 0.177	0.344 ± 0.169	24.602	0.018	0.644

OR, odds ratio; SZN, SizeZoneNonUniformity; HGLZE, HighGrayLevelZoneEmphasis; IR, InterquartileRange; SAE, SmallAreaEmphasis.

**Table 4:** Statistical results of texture features of p53 negative and positive expression groups in EC.

Texture features	Negative p53 expression	Positive p53 expression	Multivariate logistic regression analysis		AUC
			OR	*P*	
T_2_WI- texture features					
T_2_WI -wavelet-LLH_glszm_GLNN	0.021 ± 0.008	0.026 ± 0.009	57.716	0.002	0.696
T_2_WI-wavelet-HHH_firstorder_Skewness	0.058 ± 0.257	-0.054 ± 0.251	0.015	0.004	0.601
T_2_WI -wavelet-HHL_glszm_GLN	8.149 ± 3.764	7.402 ± 3.465	0.032	0.009	0.562
T_2_WI-wavelet-HHL_glcm_ClusterShade	-0.004 ± 0.013	0.004 ± 0.022	56.545	0.023	0.645
DWI- texture features					
DWI -wavelet-HLH_glszm_GLNN	0.605 ± 0.070	0.558 ± 0.054	0.008	<0.001	0.703
DWI -wavelet-LHH_glszm_GLNN	0.520 (0.501,0.571)	0.556(0.520,0.625)	20.397	0.004	0.611
DWI -wavelet-LHL_glrlm_LRLGLE	14.721 ± 5.226	19.409 ± 10.092	63.688	0.006	0.645
DWI -wavelet-LHL_glszm_SZN	2.088 (1.617,3.159)	1.588(1.000,2.582)	0.035	0.014	0.637
CE-T_1_WI- texture features					
CE-T_1_WI-firstorder_Maximum	138.960 ± 30.666	116.564 ± 33.654	0.015	0.006	0.700
CE-T_1_WI-firstorder_Kurtosis	3.265 ± 0.836	3.000 ± 0.679	0.012	0.003	0.612
CE-T_1_WI-glszm_SAE	0.219 ± 0.085	0.241 ± 0.067	38.569	0.009	0.620
CE-T_1_WI-glcm_InverseVariance	0.435 ± 0.068	0.396 ± 0.053	0.048	0.022	0.690

OR, odds ratio; GLNN, GrayLevelNon-UniformityNormalized; GLN, GrayLevelNon-Uniformity; LRLGLE, LongRunLowGrayLevelEmphasis; SZN, SizeZoneNonUniformity; SAE, SmallAreaEmphasis.

**Table 5:** The predictive performance of the models in training and validation cohort.

	Model	Training cohort			Validation cohort		
		AUC^a^ (95%CI^b^)	SEN^c^ (%)	SPE^d^ (%)	AUC^a^ (95%CI^b^)	SEN^c^ (%)	SPE^d^ (%)
Ki-67	T_2_WI model	0.741 (0.629-0.835)	53.5	85.3	0.688 (0.503-0.837)	94.1	37.5
	DWI model	0.765 (0.655-0.854)	90.7	55.9	0.691 (0.507-0.840)	52.9	87.5
	CE-T_1_WI model	0.733 (0.619-0.827)	72.1	64.7	0.651 (0.466-0.808)	94.1	43.8
	Combined^e^ model	0.922 (0.838-0.971)	90.7	82.4	0.938 (0.795-0.992)	76.5	100.0
p53	T_2_WI model	0.763 (0.653-0.853)	57.1	85.7	0.796 (0.620-0.915)	65.2	100.0
	DWI model	0.805 (0.699-0.887)	71.4	85.7	0.713 (0.530-0.856)	87.0	60.0
	CE-T_1_WI model	0.781 (0.673-0.868)	57.1	92.9	0.657 (0.471-0.812)	73.9	70.0
	Combined^e^ model	0.901 (0.811-0.957)	83.7	85.7	0.922 (0.773-0.986)	100.0	80.0

a AUC, area under the curve;

b CI, confidence interval;

c SEN: sensitivity;

d SPE, specificity;

e Combined, ADC value combined with T_2_WI + DWI + CE-T_1_WI.

### Efficacy of Five Models in Predicting the Expression Levels of Ki-67 and p53 in EC

**Figure 4:**
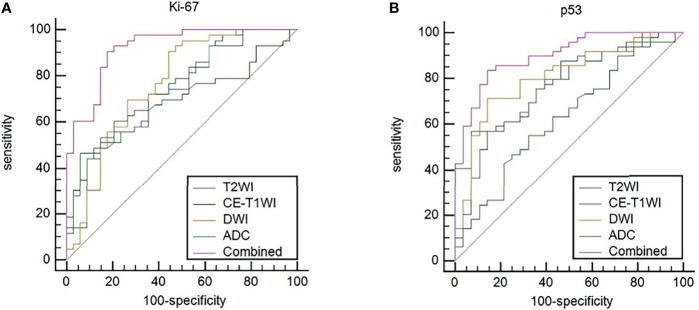
**(A)** ROC curves to predict Ki-67 expression levels in EC. **(B)** ROC curves to predict p53 expression levels in EC. Equality of AUC was assessed by the DeLong’s test.

**Figure 5:**
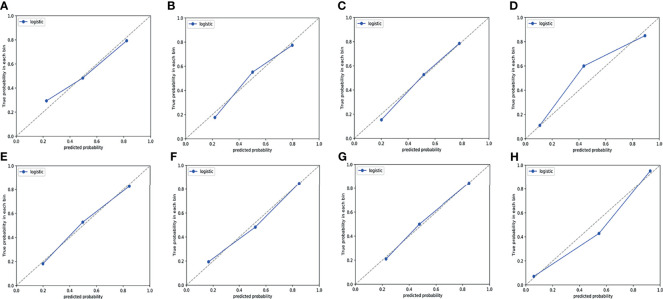
Calibration curves of the prediction model in training cohort. **(A–D)** Calibration curves for a model that predicts the expression level of Ki-67. **(A)** T_2_WI. **(B)** DWI. **(C)** CE-T_1_WI. **(D)** ADC value combined with T_2_WI + DWI + CE-T_1_WI. **(E–H)** Calibration curves for a model that predicts the expression level of p53. **(E)** T_2_WI. **(F)** DWI. **(G)** CE-T_1_WI. **(H)** ADC value combined with T_2_WI + DWI + CE-T_1_WI. The 45° dotted line represents the ideal prediction, while the blue line represents the prediction performance of the prediction model. The closer the blue line is to the dotted line, the better the performance of the prediction model.

ROC curves were used to evaluate the diagnostic efficacy of prediction models based on ADC values, T_2_WI, DWI, CE-T_1_WI, and ADC values combined with T_2_WI + DWI + CE-T_1_WI in predicting the expression levels of Ki-67 and p53 in EC. The AUC of ADC values in predicting Ki-67 and p53 expression levels in the training and validation cohorts were 0.698, 0.853 and 0.626, 0.702, respectively. The AUC of the TA model based on T_2_WI, DWI, CE-T_1_WI, and ADC value combined with T_2_WI + DWI + CE-T_1_WI in the training and validation cohorts for predicting the expression of Ki-67 were 0.741, 0.765, 0.733, 0.922 and 0.688, 0.691, 0.651, 0.938, respectively, and for predicting the expression of p53 were 0.763, 0.805, 0.781, 0.901 and 0.796, 0.713, 0.657, 0.922, respectively, with great performance. DeLong’s test was used to analyze the improvement resulting from the combined model compared to the other single models in the training and validation cohorts (**Table 6**). The diagnostic efficiency of the combined model was superior to that of the single prediction models based on ADC values, T_2_WI, DWI and CE-T_1_WI in the training and validation cohorts. In general, there was no statistical significance among each single model, but the ADC values model was superior to DWI and CE-T_1_WI in the training group for p53. The ROC curves for the training cohorts are shown in **Figure 4**. The calibration curves for each training group are shown in **Figure 5**, and the statistics of the Hosmer–Lemeshow test in each group were not significant (P > 0.05), indicating good calibration.4.

**Table 6:** AUC of the five models was compared.

	Ki-67 (*P-*value)		p53 (*P-*value)	
	Training	Validation	Training	Validation
Combined *vs*. ADC	<0.001	0.069	<0.001	0.031
Combined *vs*. T_2_WI	<0.001	0.009	0.010	0.114
Combined *vs*. DWI	0.003	0.007	0.013	0.040
Combined *vs*. CE-T_1_WI	<0.001	0.006	0.007	0.054
ADC *vs*. T_2_WI	0.609	0.140	0.077	0.450
ADC *vs*. DWI	0.399	0.063	0.033	0.944
ADC *vs*. CE-T_1_WI	0.681	0.113	0.033	0.746
T_2_WI *vs*. DWI	0.771	0.975	0.585	0.545
T_2_WI *vs*. CE-T_1_WI	0.900	0.802	0.799	0.383
DWI *vs*. CE-T_1_WI	0.702	0.783	0.714	0.701

## Discussion

In recent years, research on tumor biomarkers has increased, aiming to improve the survival rate and quality of life of cancer patients. At the same time, as early detection of EC is critical for treatment, it is important to identify reliable histopathology markers to improve diagnostic accuracy and prognosis. Previous studies have found that Ki-67 and p53 may contribute to the accuracy of cytodiagnosis of different EC lesions (17). However, Ki-67 and p53 are commonly detected using immunohistochemistry, which was not only invasive, but may also be influenced by subjective factors. In the present study, a novel MRI analysis method was proposed to detect the expression levels of Ki-67 and p53 in immunohistochemistry. Not only is MRI noninvasive, but it adds texture features without increasing the patient^’^s risk of side effects. Quantitative analysis of the texture of the image may also have an advantage over more targeted biopsies.

The ADC values reflect the diffusion of water molecules in the gap between tumor tissues (18), and they have been shown to be negatively correlated with the proliferation degree and cell density of tumors. The present study suggested that the ADC value in the high Ki-67 expression group was significantly lower than that in the low Ki-67 expression group in EC, which was similar to previous studies in EC (19). With the increase in the expression level of Ki-67, the proliferation activity of EC cells increases, tumors grow vigorously, cell density increases, the arrangement is closer, and the extracellular space decreases, resulting in lower ADC values.

p53 is located on chromosome 17p13-p3 and can be divided into two types, namely, wild type and mutant type, and the pathologically detectable form is mutant p53. When the expression of mutant p53 increases, it may give rise to higher cell proliferative activity, poorer differentiation, and more malignant degree in EC. The present study suggested that the ADC value in the positive p53 group was significantly lower than that in the negative p53 group in EC. Our results were expected and consistent with most previously reported data (10, 20). Although our study showed that the ADC value differed between different subgroups of Ki-67 and p53, the AUC of ADC value for Ki-67 and p53 were 0.698 and 0.626, respectively, which indicated poor performance. This may be because ADC values may not only be related to the movement of water molecules, but also affected by the microcirculation of the blood and tumor heterogeneity; therefore, they cannot truly reflect the movement of water molecules in tissues.

TA is a method that quantifies pixel intensity variations (heterogeneity). TA quantifies tumor heterogeneity by calculating the grey changes of pixels in the image (21). Some studies (22, 23) have found that magnetic resonance imaging–based texture features show the association among deep myometrial invasion, lymphovascular space invasion, and histological high-grade EC. However, only first-order statistical features were extracted and analyzed in these previous studies. In the present study, comprehensive texture features, including first-order statistics, shape-based, GLCM, GLRLM, and GLSZM were extracted based on T_2_WI, DWI, and CE-T_1_WI, and they were used to predict the expression levels of Ki-67 and p53 in patients with EC. The texture feature parameters are different in each sequence, and the area under the curve (AUC ≥ 0.7) of the “wavelet-HLL_firstorder_Skewness” of Ki-67 and the “firstorder_Maximum” and “wavelet-HLH_glszm_GLNN” of p53 are more significant. In our study, the Skewness value of the high Ki-67 expression group was higher than that of the low Ki-67 expression group, which indicated that the distribution of the tissue strength grade of the high Ki-67 expression group in EC was more disordered and heterogeneous. Skewness measures the asymmetry of the distribution of values about the mean value. A high skewness value indicates a more asymmetrical the strength grade distribution and greater heterogeneity. The maximum value represents the maximum grey level intensity within the ROI. In the present study, the maximum value of the p53-negative group was higher than that of the p53-positive group, indicating more tumor hemorrhage, secretion, and solid components in the p53-positive group of EC. GLNN measures the variability of grey-level intensity values in the image with a lower value indicating a greater similarity in intensity values. In the present study, the GLNN value of the p53-negative group was lower than that of the p53-positive group, which indicated that the ECs of the p53-positive group were more inhomogeneous and complex than those of the p53-negative group.

Different from previous studies, several studies (24, 25) on TA used one or two MRI sequences. We included T_2_WI, DWI, and CE-T_1_WI sequences of EC and established a series of models in which the ADC value and TA predicted the expression levels of Ki-67 and p53. When the ROC curve was used to evaluate the performance of the predictive model, the AUC of the TA model based on T_2_WI, DWI, and CE-T_1_WI in the training cohort for predicting the expression of Ki-67 were 0.741, 0.765, and 0.733, respectively, and for predicting the expression of p53 were 0.763, 0.805, and 0.781, respectively. The AUC performed well. The results showed that the TA of T_2_WI, DWI, and CE-T_1_WI was helpful to evaluate the expression levels of Ki-67 and p53 in EC. In three sequences of MRI, the AUC of the TA model based on DWI image texture features was higher, which was different from the results reported by Dong et al. (26) who distinguished solitary fibrous tumor/hemangiopericytoma and angiomatous meningioma base on texture feature. One explanation may be that DWI provides a better representation of the microscopic geometry of the EC tissue and the diffusion of water molecules inside and outside the cell and that the images contain more differential texture features with discriminating value. However, there was no significant difference in the TA diagnostic efficiency among the three sequences (T_2_WI, DWI, and CE-T_1_WI). It is noteworthy that the diagnostic efficiency of the combined models was better than that of the single model (*P* < 0.05). The combined model performed well in identifying the expression level of Ki-67 and p53 in EC with AUCs of 0.922 and 0.938 for Ki-67 expression and 0.901 and 0.922 for p53 expression in the training and validation cohorts, respectively. Thus, the present findings suggested that the constructed model of multiparameter MRI TA can capture higher-order interactions between data, reflect tumor heterogeneity from different aspects, and achieve better predictive efficiency. In addition, when using DeLong’s test to evaluate improvements to the combined model, the diagnostic efficiency of the combined models was better than that of the single ADC model, T_2_WI model, DWI model and CE-T_1_WI model (*P* < 0.05). The ADC value improved the diagnostic efficiency of the model, which was consistent with the results of previous studies (27) combining the ADC value with radiomics in EC.

However, there were several limitations in the present study. First, the sample size of this study was small, and it was a retrospective study, indicating potential bias in the selection of enrolled cases. Second, the ADC values were measured based on the maximum level of solid tumor components, which did not represent the overall tumor condition. Third, our model did not include EC FIGO stage, grading, or other clinical indicators. Therefore, future studies will be performed to further increase the sample size.

## Conclusions

In conclusion, a combination of ADC values and TA based on three MRI sequences were developed to provide a noninvasive method for preoperatively predicting the expression levels of Ki-67 and p53 in EC. To some extent, this noninvasive imaging marker can compensate for the limitation of endometrium curettage biopsy and adverse impact of tumor heterogeneity, and it provide an objective imaging basis for clinical and accurate individualized treatment.

## Data Availability Statement

The original contributions presented in the study are included in the article/supplementary material. Further inquiries can be directed to the corresponding author.

## Ethics Statement

The studies involving human participants were reviewed and approved by the Medical Ethics Committee of Anhui Provincial Cancer Hospital (2021-54). The patients/participants provided their written informed consent to participate in this study. Written informed consent was obtained from the individual(s) for the publication of any potentially identifiable images or data included in this article.

## Author Contributions

JD, XJ, HJ, CW, and CBW contributed to conception and design. XJ, HJ, and ZZ contributed to the collection and arrangement of data. XJ, HJ, CW, CBW, and JD contributed to data analysis and manuscript writing. All authors contributed to the article and approved the submitted version.

## Funding

This work was supported by 2020 SKY Image Research Fund (No. Z-2014-07-2003-11) and National key research and development program (No. 2016YFB1000905).

## Conflict of Interest

The authors declare that the research was conducted in the absence of any commercial or financial relationships that could be construed as a potential conflict of interest.

## Publisher’s Note

All claims expressed in this article are solely those of the authors and do not necessarily represent those of their affiliated organizations, or those of the publisher, the editors and the reviewers. Any product that may be evaluated in this article, or claim that may be made by its manufacturer, is not guaranteed or endorsed by the publisher.
